# Supplementary material for: Motivation and determinants of research careers among physicians: Exploring pros and cons of the Dutch MD-PhD model
Source: PLoS One. 2026 Mar 2;21(3):e0343064. doi: 10.1371/journal.pone.0343064 (PMC12952570; doi:10.1371/journal.pone.0343064)
Supplement: S1 File — (PDF) [file pone.0343064.s001.pdf]

# S1 File. English version of full survey.

## Survey on motivation and determinants of research careers among physicians in the Netherlands

* *Original survey in Dutch*

** *Survey contains conditional logic; not all respondents answered all questions*

### Demographic data

1. What is your age?

2. What is your gender?
   - Male
   - Female
   - Other
   - Prefer not to say

### PhD trajectory

3. At which university did you complete your PhD?
   - Erasmus University Rotterdam
   - Maastricht University
   - Radboud University Nijmegen
   - University of Groningen
   - Leiden University
   - Utrecht University
   - University of Amsterdam
   - Vrije Universiteit Amsterdam

4. In which field did you complete your PhD (e.g., gynaecology, general practice, epidemiology)?

5. What was your position when you started your PhD trajectory?
   - Medical student (with or without MD-PhD program)
   - Junior doctor without work experience in patient care
   - Junior doctor with work experience in patient care
   - Resident in training to become an intramural specialist
   - Resident in training to become an extramural specialist
   - Specialist (intramural or extramural)
   - Other, namely:...

6. Can you briefly explain how came to start your PhD trajectory?

**Motivation for a PhD**

7. To what extent did the following reasons or goals influence your decision to pursue a PhD or to complete your PhD trajectory?

   *(Answers on a 5-point Likert scale with 1 = fully disagree, 5 = fully agree)*

   - For the satisfaction I feel when I surpass myself in my learning activities (e.g., work, presentations).
   - For the satisfaction I have in facing challenges in my studies.
   - For the pleasure of doing research
   - Because doctoral studies are consistent with my values (e.g., curiosity, ambition, success).
   - Because my doctoral studies are a fundamental part of who I am and my identity.
   - Because my doctoral studies met my goals and my objectives in life.
   - Because I wanted to improve my skills in my field of study.
   - Because it was important for me to advance knowledge in my field of study.
   - Because I had the opportunity to take my first steps in research (e.g., publications, collaborations) while benefitting from supervision.
   - Because my supervisor would have been disappointed or angry if I gave up.
   - Because I made commitments that I must fulfil (e.g., with funding agencies, employers, collaborators, a research director).
   - Because I did not want to be perceived as a quitter.
   - For the prestige associated with a PhD.
   - To find a job with good working conditions.
   - To get a better paying job after graduation.

   - To increase the chances of securing a place in a residency program

8. Did any factors other than those mentioned above play a role in your decision to start or continue your PhD trajectory?
   - No
   - Yes, namely…

9. At the time of your PhD, did you intend to remain scientifically active in your future career?
   - Yes
   - No

10. Please elaborate on your answer to question 9.

11. Have you remained scientifically active after completing your PhD (whether or not after a break, for example, for a medical specialty training)?

12. Which factors contributed to you remaining scientifically active after completing your dissertation? (Multiple answers possible)
    - Motivation for conducting scientific research
    - Scientific network or collaboration opportunities
    - Protected time for conducting scientific research
    - Availability of sufficient research funding
    - Adequate preparation for independently conducting scientific research
    - Support from supervisors
    - Support from colleagues
    - Support from family and/or friends
    - Other, namely...

13. What type of research do you conduct?
    - Biomedical research
    - Clinical research
    - Both biomedical and clinical research
    - Other, namely...

14. Approximately how many new articles have you published since defending your dissertation?

15. Which factors contributed to you not remaining scientifically active after completing your PhD?
    - Lack of motivation for conducting scientific research
    - Lack of scientific network or collaboration opportunities
    - Lack of protected time for conducting scientific research
    - Lack of research funding
    - Lack of support from supervisors
    - Inadequate preparation for independently conducting scientific research
    - More time for partner/family
    - More time for other activities outside of work
    - Other, namely...

16. Did you pursue medical specialty training?
    - No
    - Yes, namely…

17. Where are you currently employed?
    - Academic hospital
    - Top clinical hospital
    - General hospital
    - General practice
    - Private clinic
    - Industry

- Other, namely...

18. Do you experience advantages in your clinical work due to having a PhD compared to colleagues who do not have a PhD?
    - Yes
    - No
    - I no longer have clinical duties

19. In what areas do you experience these advantages? (Multiple answers possible)
    - In daily patient care
    - Application of Evidence-Based Medicine in the clinic
    - Development of guidelines and/or protocols
    - Conducting and/or coordinating clinical research
    - Other, namely...

20. Have you experienced any disadvantages in your career as a result of your PhD trajectory?
    - Yes
    - No

21. Please elaborate on your answer to question 20.

22. How many hours per week does your current contract specify you work?

23. How many hours per week do you actually work on average in your current job?

24. What percentage of your total actual working time do you typically spend on...
    - …clinical work?
    - …research?
    - …education?
    - …management?
    - …other tasks?

25. What is your current academic rank?
    - None
    - Postdoc
    - Assistant professor
    - Associate professor
    - Full professor
    - Other, namely…

26. If you could go back in time and choose again, would you start a PhD trajectory?
    - Yes, at the same time and in the same specialty

- Yes, at the same time but in a different specialty
- Yes, in the same specialty but at a different point in my career
- Yes, at a different point in my career and in a different specialty
- No

27. Please elaborate on your answer to question 26.

28. How satisfied are you with your career progression so far?
*(Answer on a 5-point Likert scale with 1 = dissatisfied, 5 = very satisfied)*

29. Please elaborate on your answer to question 28.

30. Do you think you could have reached your current position without your PhD trajectory?
*(Answer on a 5-point Likert scale with 1 = very unlikely, 5 = very likely)*

31. Do you have any additional comments regarding the topic of this questionnaire?
