# Supplementary material for: Motivation and determinants of research careers among physicians: Exploring pros and cons of the Dutch MD-PhD model
Source: PLoS One. 2026 Mar 2;21(3):e0343064. doi: 10.1371/journal.pone.0343064 (PMC12952570; doi:10.1371/journal.pone.0343064)
Supplement: Attachment — Submitted filename: Response to Reviewers.docx [file pone.0343064.s003.docx]

|  | **Reviewers’ comment** | **Description of revisions** | **Location of revisions**  NB: page and line numbers match with the document with track changes |
| --- | --- | --- | --- |
|  | **Reviewer #1** | | |
|  | PLoS ONE is an international journal, and the article will certainly be read by many scientists and educators outside the Netherlands. I therefore suggest that the authors describe the Dutch Medical education system in greater detail. Here are a few suggestions: |  |  |
| 1. | Does the MD degree require a research thesis (as for example in Germany or Switzerland) or is it granted «automatically» together with the Medical degree (as for example in the US and in Austria). | In the Netherlands, the MD degree does not require a research thesis. However, a research project resulting in a research report is mandatory in all curricula. We have expanded the Context section with a brief description of the MD degree in Netherlands. | Methods, Context section  page 7, lines 96-105 |
| 2. | The authors speak of PhD not MD-PhD programs. Are these PhD programs specifically designed for and open only to health professionals? Which health professionals can enter the programs? What is the percentage of MDs versus other health professionals entering the PhD programs. | In the Netherlands, PhD candidates are not students, but employees. This is the case for PhD candidates in all domains. Therefore, there is no group of students that start the same program at the same time, as is the case in for example MD-PhD programs in other countries.  We have expanded the Context section with a description of the PhD trajectory in the Netherlands. | Methods, Context section  page 8-9, lines 114-134 |
| 3. | How do the health professional PhD programs compare to regular PhD programs at Science faculties. | The employee status described above applies to all PhD candidates in the Netherlands. While the specific graduate school or training program may differ between disciplines, the overall structure of the PhD trajectory is essentially the same across disciplines.  Therefore, no further changes were made to the manuscript other than the abovementioned additions to the Context section on pages 6 and 7. | - |
| 4. | Does the Dutch system have a habilitation, as it is known in many German speaking countries? If so, does the MD-PhD substitute for the habilitation. | The Dutch system has no habilitation. In the Netherlands, the PhD degree is the highest academic degree. After obtaining the PhD degree, PhD graduates who wish to continue their research or pursue an academic career typically apply for postdoctoral research positions. This was also specified in the description of the PhD trajectory in the Context section. | Methods, Context section  pages 8-9, lines 131-134 |
|  | Other points: |  |  |
| 5. | The sub-optimal return rate of the questionnaires is an inherent problem to almost all of these surveils. Persons working in top Dutch institutions seem over-represented among the respondents. That likely introduces a bias and should be discussed. | In general, the response rate can be problematic for a survey study. However, our response rate of 56.6% is relatively high compared to many other survey studies, and sufficient (>50%) for reporting relevant outcomes. We have added a suboptimal response rate to the limitations section on page 25.  We do not feel that the overrepresentation of persons working in top Dutch institutions introduces a bias. The full cohort includes MD-PhDs only, who are generally more likely to end up in academic or top-clinical hospitals. Moreover, a previous study (Introduction page 5 line 70, reference 20) that investigated the current positions of the same cohort based on the affiliations of MD-PhDs found that 43% of the MD-PhDs held a position in an academic hospital. In our survey results, 43.5% of respondents indicated to work in an academic hospital. The respondents therefore seem to be a good reflection of the full cohort. | Discussion, Limitations section, page 27, lines 522-523 |
| 6. | Some recent literature on MD-PhD programs in other European and non-European countries should be discussed: e.g. PMID: 38980543; PMID: 40036075; PMID: 39704177; PMID: 39240708; PMID: 39103197; PMID: 38958475; PMID: 38917318; PMID: 38329127 | Thank you for suggesting these references. We especially found PMID: 38980543 an interesting reference, and have included this reference in our discussion to compare the results of our study to the results of the Swiss cohort of MD-PhDs described in this study.  While the other studies do address MD-PhDs, we felt they did not directly relate to the focus of our study, for example by focusing on how application metrics for MD-PhD programs predict physician-scientist outcomes or comparing the financial impact of MD-PhD vs MD training. We therefore chose not to incorporate the other suggested references, to maintain to focus of our manuscript. | Discussion, page 24-25, lines 467-474 |
|  | **Reviewer #2** | | |
|  | Abstract |  |  |
| 7. | First, there is an error in the SDT terminology. In the Results subsection of the abstract, autonomous motivation and intrinsic motivation are conflated. Controlled motivation and extrinsic motivation are also conflated. According to SDT, (1) autonomous motivation can be derived from intrinsic motivation and internalized forms of extrinsic motivation and (2) controlled motivation is a version of extrinsic motivation but not all extrinsic motivation is controlled. Please revise accordingly. | This statement was indeed incorrect. We have changed it to a statement that is SDT correct. | Abstract Results section, page 3, lines 17-19 |
| 8. | In the Method section, on Page 7, starting on Line 126, you begin to describe the Motivation for PhD Studies Scale. In the abstract, it was not clear that the only measure of SDT included in your paper would be this scale (i.e., “We used self-determination theory and the Motivation for PhD Studies Scale…”). Please consider revising this statement to something such as “Drawing on self-determination theory, we used the Motivation for PhD Studies Scale…” to limit confusion for the reader. | We have followed your suggestion and revised the abstract accordingly. | Abstract Methods section, page 3, lines 11-12 |
|  | Introduction |  |  |
| 9. | On Page 5, paragraph 1 begins with a focus on “concerns about the effectiveness of the Dutch [MD-PhD] system”. However, I noticed that some of the citations in this paragraph are not actually about the Dutch MD-PhD system. For example, while citation #17 pertains to a relevant issue–the Publish or Perish crisis–the data is based solely on US MD students. Please be more clear about whether the papers you are citing are specifically based on Dutch data or were conducted with international data from which you are generalizing. | We have removed the references that were not based on Dutch data (2 references). We have added 1 new reference that refers to the quality of research (Huisjes M. Promoveren werkt. Amsterdam: Koninklijke Nederlandse Akademie van Wetenschappen; 2016.). | Introduction section, page 6, lines 62 and 66 |
|  | Methods |  |  |
| 10. | In the Context subsection, the type of PhD programs awarded by the eight UMCs are not specified. Are all of the PhD programs of medical and biomedical science programs? Those are the only kinds referenced in this section. It would be helpful if you specify what type(s) of PhDs were earned by the MD-PhDs in your sample and if this is representative of all types of PhDs earned by graduates of MD-PhD training pathways. | In the Netherlands, PhD candidates are considered researchers, not students. Most PhD candidates are employed by a university in a paid research position for the duration of the PhD trajectory. Applying for a PhD position is therefore similar to applying for a regular job vacancy, rather than enrolment in tuition-paying student cohorts. Therefore, there is no group of students that start the same program at the same time, as is the case in MD-PhD programs in other countries. In the Netherlands, PhD candidates do usually follow doctoral education at a university graduate school where they must be registered. While there is no distinction between PhD candidates between different domains, the type of experiments or graduate education may differ depending on the research domain.  We have expanded the Context section with a description of the PhD trajectory in the Netherlands. | Methods, Context section  page 8-9, lines 114-134 |
| 11. | On Page 8, you write “We conducted 4 cognitive interviews…” but do not specify with whom the cognitive interviewing was conducted. Was this also done with MD-PhDs who obtained their PhD in a year other than 2008? On a related note, though I assume this is the case, you do not state if the cognitive interviewing and pilot testing was conducted with Dutch MD-PhD recipients and if the interviewing and testing was done in Dutch. Please clarify. | The cognitive interviews were conducted by two of the authors with Dutch MD-PhDs who obtained their PhD degree around but not in 2008. The pilot group also consisted of Dutch MD-PhDs. We have clarified this in the text on page 9. | Methods, Survey development and data collection section, page 10, lines 174-182 |
| 12. | On Page 8, you note that the complete survey is available as Supplemental Digital Appendix 1. Thank you for providing this. As a reader, I needed more detail regarding the survey measures in the main paper. For example, it was unclear to me that career satisfaction was measured until I read the results section. Similarly, a brief description of what content was being coded for would be helpful (see Page 9, narrative data was content-coded by two of the authors). Without additional information, it is unclear to the reader what content domains were gleaned from the open-ended questions. Please provide more detail. | We have added career satisfaction to the description of the survey in the main text on page 8 (Methods, Survey development and data collection section).  We have also added an explanation of the narrative content that was coded (Methods section, page 11). | Methods, Survey development and data collection section, page 10, line 157  Methods, Data analysis section, page 13, lines 236-246 |
| 13. | You translated the Motivation for PhD Studies Scale to Dutch from another language. I strongly urge you to provide more detail regarding the translation. What was the original language? Who translated the measure, and did they use a specific method such as back-translation to ensure the translation was as intended? Was this partially the purpose of the cognitive interviews and pilot testing? Most importantly, given the translation to a new language, a confirmatory factor analysis (CFA) is warranted. This would provide reassurance that the translations did not change the internal loading structure of the items into the two overarching categories of autonomous and controlled motivation. | We have translated the MPhD scale from English to Dutch. In the text we have clarified this, and we also added who was responsible for the translation.  We did not conduct a formal back-translation because the questionnaire required context-specific wording adaptations to fit the target population and to assess retrospective rather than current motivation. We therefore knew that a literal back translation would come back with different wording than the original MPhD scale. Part of the purpose of the cognitive interviews and pilot testing was indeed to see whether the respondents’ interpretation of the items matched our intentions. We have also specified this in the main text.  In addition, for validation we have now added the results of our SEM to the manuscript. | Methods, Survey development and data collection section, page 10, lines 166-174  Methods, Survey development and data collection section, pages 10-11, lines 174-182.  Methods, Data analysis section, page 12, lines 205-213  and  Results, Motivation to pursue a PhD section, pages 14-15, lines 259-266 |
| 14. | As noted on Page 9, multiple data transformations were employed (e.g., overtime hours and published articles were transformed using the natural log. Please provide a brief rationale for any transformations. Similarly, a brief explanation on how to interpret Odds-Ratios would greatly increase the accessibility and readability of your results section. | Variables with a skewed distribution were natural log transformed to obtain a roughly normal distribution of the data. This was specified in de the Methods, Data analysis section, as well as an explanation of how to interpret the ORs of transformed variables.  In the results section we have also added a brief interpretation of the ORs for clarification. | Methods, Data analysis section, pages 12-13, lines 226-232.  Results, Physician-scientist careers section, page 18, lines 324-331, and Results, Academic tenure section, page 20, lines 360-366 |
|  | Results |  |  |
| 15. | It is not clear to the reader how many respondents in your analysis had entered a physician-scientist career. A flow chart would be extremely helpful. Your sample started with 1,034 PhD graduates from 2008. Of those, 479 were MD-PhD holders. Of those, how many were you able to contact either directly or indirectly? Of those, 240 responded to your survey. Of those, 60 were deemed to be physician-scientists. Given that some analyses include all respondents while others only include a subgroup (mostly the physician-scientists), it is important to be very detailed about the sample size. The tables are mostly clear; the write-up is more inconsistent. | We have added numbers of respondents to the percentages mentioned, to help the reader understand the sample sizes. | All throughout Results section on pages 17-22 |
| 16. | Building on Point #10, throughout the Results section, the sample size of each analysis/subgroup is not consistently highlighted. For example, the Ns for each correlation are missing in Table 2. The same issue applies to Table 4, where it is particularly important to note the sample size as the sample at this point is restricted to an upper bound of only 60 respondents. | We have added the sample sizes where they were lacking in Table 2 and Table 4.  For Table 4, the sample sizes refer to the total number of respondents included in each model. While the number of physician-scientists is restricted to 60, the number of respondents in each model is restricted to 240 and depends on missing data. The reported odds ratios reflect the odds of pursuing a physician-scientist career versus not pursuing such a career across the full sample. The total sample size contributing to the odds ratios can therefore exceed 60. We have clarified the sample size for each model in Table 4. | Results, Table 2, page 16, and Table 4, page 19 |
| 17. | Also building on the prior two points, percentages are not consistently used throughout the Results section. For example, the Advantages of PhD in clinical work subsection provides percentages to indicate the frequency of each of the stated types of advantages, yet the Disadvantages of the PhD and Career satisfaction subsections does not follow the same format. I consider the percentages to be very important, as the sample size is relatively small and terms such as “quite often” or “some respondents” are subjective and vague. | We have changed the text and added the number of respondents to the percentages for clarity. We also changed the wording so that the paragraphs describing the advantages and disadvantages are consistent, and we have replaced terms like ‘quite often’ with the actual number of respondents. | Results, page 21-23, lines 371-419 |
| 18. | A general suggestion: consider reorganizing the results section so that the results proceed from the largest sample possible (all respondents) to the smallest sample (physician-scientists). This would make it easier for the reader to follow which analyses are being conducted with which portion of the sample. | We have implemented all previous suggestions aimed at improving readability and clarifying the sample size for each section. We did not further reorganize the Results section, as we believe that presenting the findings thematically also is helpful for the reader to understand the results within each section. | - |
| 19. | On Page 15, you state “Autonomous motivation was a significant predictor of achieving academic tenure.” Was controlled motivation not a significant predictor? Even if the result is not deemed statistically significant, the result should be reported. | We have added the results for controlled motivation. | Results, Academic tenure section, page 20, lines 362-363 |
|  | Discussion |  |  |
| 20. | On Page 18, you state “...findings are in line with a recent survey[28] among current medical PhD students.” While the study is recent relative to now, it likely would not be perceived that way in another 10 years when people are hopefully still reading your paper. I suggest you state the year, as recent is subjective. | We have added the year of the survey. | Discussion, page 24, line 451 |
| 21. | On Page 18, please specify that NHS stands for the UK’s National Health Service. | We have specified that NHS stands for the UK’s National Health Service. | Discussion, page 25, line 476 |
| 22. | In the Discussion, I felt there was a missed opportunity to callback to a point you made in the Introduction–autonomous motivation is more beneficial than controlled motivation for work performance and persistence. Without this connection, the reader is left asking “but why did we measure these aspects of motivation?” and “how does this extend the SDT literature?” I was surprised that your Discussion does not have a clearly defined section for theoretical contributions, and I urge you to include one. | In the Discussion, we have now made a clear link between our results and SDT. | Discussion, page 23-24, lines 432-433 and lines 437-449 |
| 23. | The final Conclusions subsection feels a bit disjointed from the rest of the Discussion. While the Discussion does have a section on Page 18 that highlights differences in the sizes of MD-PhD populations in the Netherlands, UK, and United States, it does not set up the concluding statement that “Future studies could explore whether further increasing numbers of MD-PhDs internationally could help increase the physician-scientist workforce.” (Page 21) Furthermore, it is not immediately clear to the reader why we should increase the physician-scientist workforce, as that brief point from Page 4 of the Introduction has not been revisited in the Discussion. | We have revisited the need for physician-scientists in the Discussion.  In the Conclusion, we have added how our results suggest that the proportion of physician-scientists among Dutch physicians may be slightly higher than in both the US and the UK, to clarify why future prospective studies could explore whether this is indeed the result from a higher proportion of MD-PhDs. | Discussion, page 23, lines 421-424  Conclusion, page 28, lines 544-549 |
